# Monitoring *Mycoplasma bovis* Diversity and Antimicrobial Susceptibility in Calf Feedlots Undergoing a Respiratory Disease Outbreak

**DOI:** 10.3390/pathogens9070593

**Published:** 2020-07-21

**Authors:** Claire A.M. Becker, Chloé Ambroset, Anthéa Huleux, Angélique Vialatte, Adélie Colin, Agnès Tricot, Marie-Anne Arcangioli, Florence Tardy

**Affiliations:** 1 UMR Mycoplasmoses des Ruminants, VetAgro Sup, Université de Lyon, 69280 Marcy-l’Etoile, France; chloe.ambroset@vetagro-sup.fr (C.A.); agnes.tricot@vetagro-sup.fr (A.T.); marie-anne.arcangioli@vetagro-sup.fr (M.-A.A.); 2 UMR Mycoplasmoses des Ruminants, ANSES Laboratoire de Lyon, Université de Lyon, 69364 Lyon CEDEX 07, France; anthea.huleux@orange.fr (A.H.); angelique.vialatte@gmail.com (A.V.); adelie.colin@anses.fr (A.C.); florence.tardy@anses.fr (F.T.)

**Keywords:** *Mycoplasma bovis*, antimicrobial resistance, Bovine Respiratory Disease, genetic diversity

## Abstract

Bovine respiratory diseases (BRD) are widespread in veal calf feedlots. Several pathogens are implicated, both viruses and bacteria, one of which, *Mycoplasma bovis*, is under-researched. This worldwide-distributed bacterium has been shown to be highly resistant in vitro to the main antimicrobials used to treat BRD. Our objective was to monitor the relative prevalence of *M. bovis* during BRD episodes, its diversity, and its resistance phenotype in relation to antimicrobial use. For this purpose, a two-year longitudinal follow-up of 25 feedlots was organized and 537 nasal swabs were collected on 358 veal calves at their arrival in the lot, at the BRD peak and 4 weeks after collective antimicrobial treatments. The presence of *M. bovis* was assessed by real-time PCR and culture. The clones isolated were then subtyped (*polC* subtyping and PFGE analysis), and their susceptibility to five antimicrobials was determined. The course of the disease and the antimicrobials used had no influence on the genetic diversity of the *M. bovis* strains: The subtype distribution was the same throughout the BRD episode and similar to that already described in France, with a major narrowly-variable subtype circulating, st2. The same conclusion holds for antimicrobial resistance (AMR) phenotypes: All the clones were already multiresistant to the main antimicrobials used (except for fluoroquinolones) prior to any treatments. By contrast, changes of AMR phenotypes could be suspected for Pasteurellaceae in two cases in relation to the treatments registered.

## 1. Introduction

Bovine Respiratory Disease (BRD), also known as “shipping fever”, is a very common and extremely costly disease impacting the beef cattle industry worldwide [1]. It is a complex viral and/or bacterial infection affecting the upper or lower respiratory tracts in cattle, with a particularly high prevalence in recently weaned calves within the first days or weeks of arrival at the feedlot [2]. Multiple stress factors (weaning, transportation, co-mingling in lots or in markets, changes in diet, weather changes, etc.) with additive effects are known to influence the susceptibility of calves to developing BRD [3]. The percentage of morbidity and mortality can reach 70% but varies with the management system in place, prevention programs and the kind of pathogens involved, bacteria being more often fatal than viruses alone [1]. The disease most often results from an overwhelming, dysregulated host immune response [4]. Classical clinical signs of bacterial BRD include fever over 40 °C, dyspnea, nasal discharge, coughing and depression with diminished or no appetite [2]. The most common viral agents associated with BRD include Bovine Herpes Virus type 1 (BHV-1), Parainfluenza-3 virus (PI3), Bovine Viral Diarrhea Virus (BVDV), Bovine Coronavirus (BCoV) and Bovine Respiratory Syncytial Virus (BRSV). The main bacteria are *Mannheimia haemolytica*, *Pasteurella multocida*, *Histophilus somni* and *Mycoplasma* (*M*.) *bovis* [5,6]. These agents are not all equivalent in terms of pathogenesis, duration of the clinical disease or shedding after exposure [5,6]. Viral agents are thought to be mainly initiators of the disease that then facilitate colonization by bacterial pathogens or aggravating factors during co-infection [7]. Among bacteria, *M. bovis* is still regarded as the least well-characterized BRD pathogen [6,7,8]. It has been reported to rapidly proliferate in the nasopharynx within the first 14 days of feedlot placement as a preliminary step in the development of BRD [3,9]. Asymptomatic carriage months or even years after an outbreak have been described but with a low prevalence and a role on transmission yet to be defined [10].

Prevention and control of BRD rely on metaphylaxis in high-risk herds (e.g., >1000 animals in the USA), bacterial vaccinations when available, but with controversial efficacies, and antimicrobial treatments of diseased animals [11,12]. Because feedlot management uses many antimicrobials, antimicrobial resistance (AMR) among the bacterial pathogens commonly associated with BRD has been increasingly reported worldwide [12,13]. *M. bovis* is no exception [14]. 

In France, previous studies have demonstrated the spread of an *M. bovis* clonal population with acquired resistance to most antimicrobial families except for fluoroquinolones [15,16,17]. However, these data capture a particular context of sampling that might not reflect the short-term evolution of isolates toward AMR. All these studies used strains collected in the framework of our network, Vigimyc, a “passive” surveillance network, the decision to test for mycoplasmas being solely on the initiative of the veterinarian [18]. Most often, a diagnosis for *Mycoplasma* is requested when all other analyses have proved negative or when a treatment failure is observed. Biased sampling might therefore result from using Vigimyc strains as they often originate from antimicrobials-treated animals. *Mycoplasma* species are known to evolve fast, and they develop AMR mainly through mutations in antimicrobial targets, which could be rapidly selected under antimicrobial pressure [19,20,21,22]. 

The present study was conducted to refine our understanding of relationships between antimicrobial use, AMR phenotype (as Minimum inhibitory concentration, MIC) and clonal diversity in *M. bovis* during BRD episodes. For that purpose, a longitudinal follow-up of 25 feedlots was conducted, with complete etiological exploration when BRD cases occurred, from the day of introduction to 4 weeks after the clinical peak. *M. bovis* isolate diversity per feedlot and per animal (by molecular subtyping and Pulsed-Field Gel Electrophoresis, PFGE) and AMR were analyzed before and after treatments. The relative persistence of *M. bovis* and Pasteurellaceae after antimicrobial treatment was also explored.

## 2. Results

### 2.1. M. bovis Was the Third Most Frequently Isolated Pathogen (in Association with Others) in Calf Feedlots at BRD Onset

**Figure 1:**
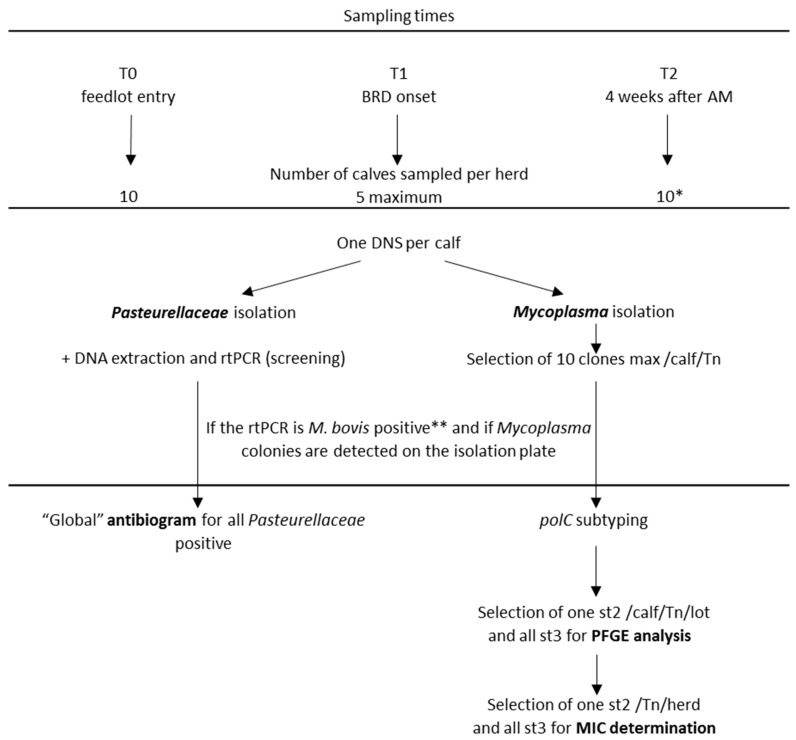
Workflow for sample collection and analyses. DNS, double nasal swab; AM, antimicrobial treatment. * If possible the same 10 calves were sampled at T0 and T2. ** Ct ≤ 37 in rtPCR.

During the 2016–2017 and 2017–2018 winters, 537 double nasal swabs (DNS) were sampled on 358 veal calves in 25 feedlots of Western France. Their characteristics are listed in Supplementary Table S1. Three sampling times were defined: introduction of the animals (T0), BRD peak (T1) and 4 weeks after collective antimicrobial treatment (T2) (Figure 1, Table 1). Four feedlots were excluded from the prevalence study as no BRD episode occurred.

**Figure 2:**
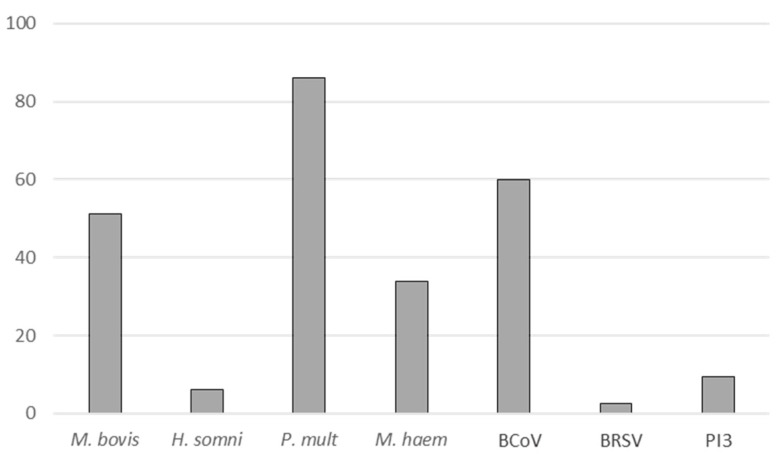
Proportions of calves harboring the different pathogens as assessed by rtPCR at BRD onset (T1, *n* = 115 calves tested).

At BRD onset (T1), the etiology was determined using a real-time Polymerase Chain Reaction (rtPCR) screening of seven pathogens (Figure 2). Out of the 21 feedlots where BRD occurred, 115 calves were tested, and *M. bovis* was detected in 51% of them (*n* = 59), with a mean Ct of 25.7 [20.4–35]. The positive calves (Ct ≤ 37) originated from 18 feedlots: Three feedlots had only calves negative for *M. bovis* (Supplementary Table S1). *M. bovis* ranked as the third most prevalent pathogen after *P. multocida* and the Coronavirus, this triple association being the most common coinfection, calves being frequently infected by more than one pathogen. The proportion of calves found positive at T1 by a culture approach was very similar to that from rtPCR (52%, *n* = 60) (Table 1). We note that for some calves in different lots (e.g., ME, Supplementary Table S1), cultures were initially positive for *M. bovis*, but no clones could be successfully retrieved from plates, mainly due to coinfections with *M. bovirhinis*. By contrast, *M. bovis* was seldom detected at feedlot entry (T0). Detection rate was 2%: 5/271 calves by culture and 6/271 by rtPCR, with a mean Ct of 36.7 [25.4–36.8], this difference being compatible with a weak infectious load in the positive calves. Four weeks after the antimicrobial treatment (T2), the presence of *M. bovis* remained high as determined by rtPCR (49%, 39/79, 12/13 feedlots being positive (Supplementary Table S1) with a mean Ct of 30.4 [23.6–36.7]. These Ct values suggest that at this stage of clinical recovery, the viable *Mycoplasma* load was rather low, as the proportion of calves positive by culture was only 17% (25/151 calves, 13/24 lots being considered positive, see Supplementary Table S1). 

The different pathogens tested, indicated on x-axis, are *Mycoplasma bovis* (*M. bovis*), *Histophilus somni* (*H. somni*), *Pasteurella multocida* (*P. mult*), *Mannheimia haemolytica* (*M. haem*), Bovine Coronavirus (BCoV), Bovine Respiratory Syncytial Virus (BRSV) and Parainfluenza 3 virus (PI3). Y-axis, percentage of calves infected with each pathogen.

### 2.2. The Diversity of Clones Does Not Differ at Different Sampling Times and Is Similar to the Reference Population of Vigimyc Isolates 

**Figure 3:**
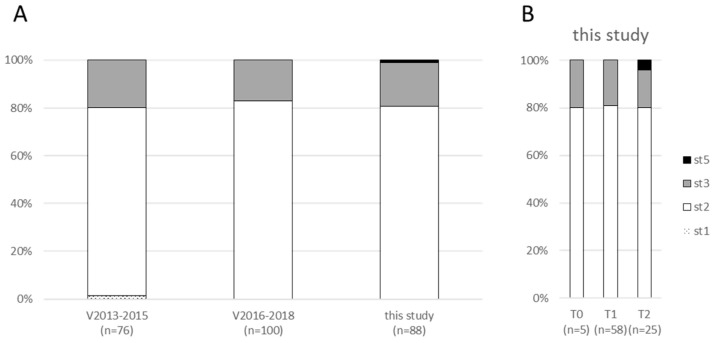
Subtype proportions of French strains of the Vigimyc network over the past 6 years and the clones of this study determined by *polC* subtyping. (**A**) Global comparison between Vigimyc strains and those from the present study and (**B**) detailed proportions of st according to sampling times (T0, T1, T2) in this study. X-axis, category of strains (Vigimyc, V, with different sampling year or this study); y-axis, proportion of each subtype. Numbers in brackets under each lane indicate the number of strains tested.

The number of isolated clones per feedlot (with a maximum of 10 per calf) selected from agar plates, with their characteristics, is given in Supplementary Table S1. Out of the 414 clones retrieved from the 93 calves sampled at different times in the 25 feedlots, 400 were subtyped using the *polC* subtyping scheme as proposed earlier [15]. Most (313/400, 78%) st2 was recovered, but st3 was also present (86/400, 21%). Both subtypes could be found in the same feedlot at the same sampling time, but no calf was shown to harbor both subtypes at one sampling time. For this reason, we further analyzed the proportion of subtypes per calf at each sampling time, which we considered thereafter as our epidemiological unit. This proportion was of 81% calves having a st2 *M. bovis* (Figure 3A), while 18% had a st3 *M. bovis*. Surprisingly, a new st was defined in the French scheme for one calf (T2-RO-1647, black on Figure 3) showing 15 SNPs with respect to the reference sequence of PG45^TS^ (11 out of 15 SNPs in common with the st3 sequence). The subtype determined using the MLST scheme of Register was ST45 (legacy scheme, ST124) [23,24].

The st proportions were identical between different sampling times (Figure 3B). No clear evolution of subtypes proportions was observed along the study, whatever the antimicrobials used in the herds (Supplementary Table S2). Some calves harbored the same st in T1 and T2 (e.g., HA-8680 with st2 or NE-6423 with st3), whereas others had a different st at the two sampling times (e.g., NE-8907 st2 then st3 or CA-8149 st3 then st2), suggesting a potential contamination by another strain in the course of the fattening period.

The overall proportions of the st in the study (calculated on calf numbers) was compared to the diversity retrieved among the strains of the Vigimyc network over the past six years (Figure 3A). The same epidemiological tendency was observed between the study strains and the Vigimyc strains: The st2 *polC* subtype was the most prevalent, while the proportion of st3 was similar at each sampling date in this study (19%).

**Figure 4:**
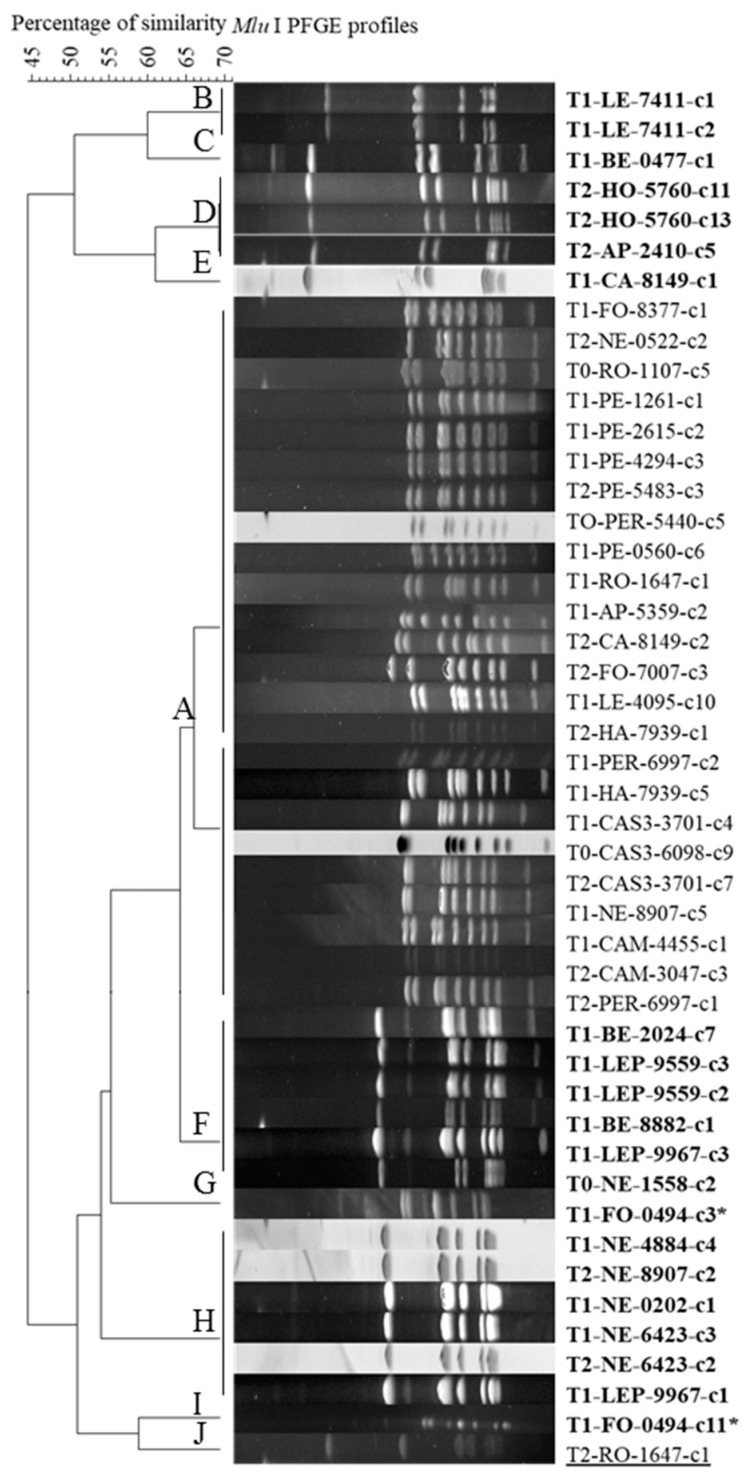
Cluster analysis of 46 *M. bovis* clones based on their Pulse Field Gel Electrophoresis (PFGE)-*MluI* profiles using the Dice coefficient and UPGMA method. The resulting degree of similarity is indicated on the scale on the top left corner (similarity cut-off value set at 70%). *M. bovis* clones are characterized by the sampling time, feedlot name, calf number and clone number. (**A**) cluster of st2 strains; (**B**–**I**) clusters of st3 strains (bold); J, branch for the clone of new st5 (underlined); the asterisk indicates 2 clones coming from the same calf with different PFGE profiles.

A restricted panel of clones was then analyzed by PFGE to further evaluate their relatedness. We first evaluated intra-st2 diversity by analyzing all the clones from two feedlots. All the st2 clones retrieved from eight sampled calves showed a unique, identical PFGE pattern, with no difference between calves or feedlots (data not shown). Consequently, in further analyses, for st2, only one clone per calf and per sampling time was selected. Because an increased diversity was expected from st3 clones [15] and as these were less numerous, they were all tested by PFGE. The PFGE patterns were homogeneous for all the st2 clones (Branch A in Figure 4) while the st3 clones showed more diversity (Branches B to I, in Figure 4, see also Supplementary Table S2). This within-st3 diversity was observed at different levels, i.e., different feedlots, different calves, or even different clones isolated from the same calf (e.g., T1-FO-0494-c3 or c11 * in Figure 4). However, no correlation was established between the PFGE profiles and the treatment history or sampling date. The st5 clone was found in a specific, different branch (J in Figure 4), showing a more distant profile. 

### 2.3. M. bovis Field Isolates Are Already Multiresistant to Antimicrobials before Any Treatment in the Lot

**Figure 5:**
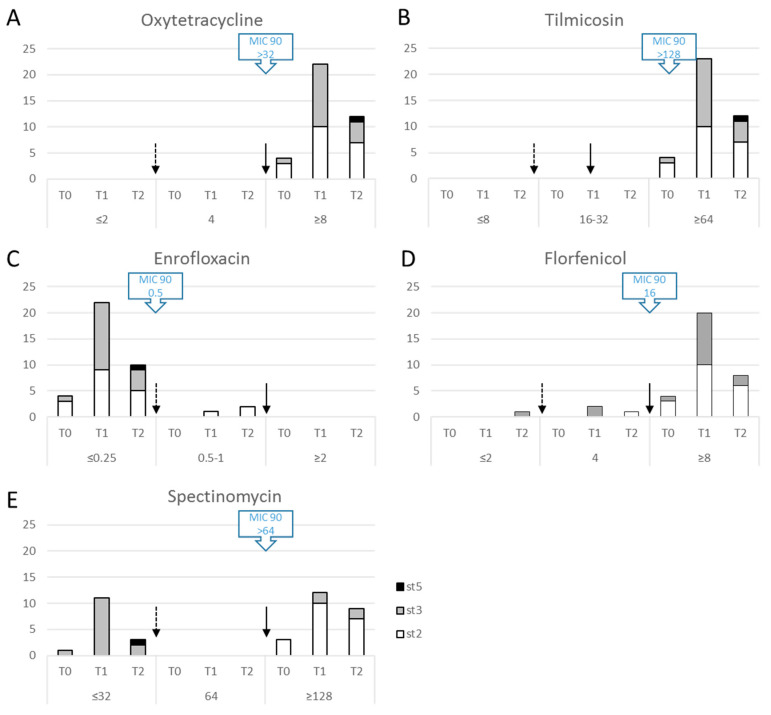
Minimum Inhibitory Concentrations (MICs) for 5 antimicrobial molecules for 39 clones according to their genetic st (*polC* subtyping) and sampling date. (**A**) Oxytetracycline, (**B**) Tilmicosin, (**C**) Enrofloxacin, (**D**) Florfenicol, (**E**) Spectinomycin. X-axis, MIC classes detailed with sampling times; y-axis, number of isolates. Arrows indicate the threshold for Pasteurellaceae when available; dotted arrow, susceptible to intermediate MIC; plain arrow, intermediate to resistant MIC. Blue rectangles with arrow indicate the MIC90 for the French *M. bovis* strains collected between 2010–2012 [16] (oxytetracycline > 32, tilmicosin > 128, enrofloxacin 0.5, florfenicol 16, spectinomycin > 64).

Antimicrobial susceptibility was tested on 39 clones representative of the various genetic subtypes or macrorestriction profiles and the different sampling times, i.e., one representative clone of st2 per feedlot and per sampling time and one representative clone per PFGE cluster per calf and per sampling time for st3 (see Supplementary Table S2). As no breakpoints are available for *M. bovis*, clinical breakpoints for Pasteurellaceae were used for interpretation of the results [25] and the MIC90 for the French *M. bovis* population (as calculated from Gautier Bouchardon et al., 2014 for recent isolates, collected between 2010 and 2012 [16]) are also indicated (blue double arrows in Figure 5). Whatever the sampling time, all the clones were resistant to oxytetracycline, tilmicosin and florfenicol (Figure 5A,B,D), except for one susceptible and three intermediary strains for florfenicol (Figure 5D). By contrast, most of the clones showed a low MIC of enrofloxacin (≤0.25 µg/mL, Figure 5C), except for three clones with a slight increase in MIC (0.5–1 µg/mL). These three clones were isolated from three different feedlots (see Supplementary Table S1): in one of these, oxolinic acid was used once to treat some calves before T1. No relationship was observed between MIC and sampling time, or with subtype.

**Figure 6:**
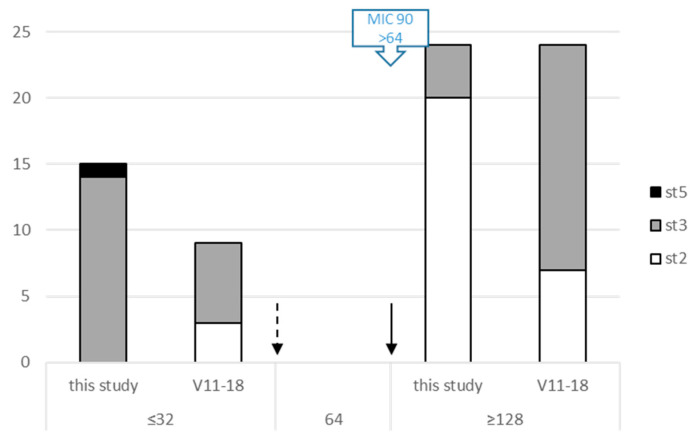
Distribution of *M. bovis* strains MICs for spectinomycin as a function of their origin and genetic subtype (*polC* subtyping). X-axis, spectinomycin MICs for sets of strains (either from Vigimyc and isolated between 2011 to 2018, V11-18, or from this study); y-axis, number of isolates. Arrows indicate the threshold for Pasteurellaceae when available; dotted arrow, susceptible to intermediate MIC; plain arrow, intermediate to resistant MIC. Blue rectangle with arrow indicates the MIC90 for the French *M. bovis* strains collected between 2010–2012 [16] (spectinomycin > 64).

For spectinomycin, 15/39 strains showed surprisingly low MICs (Figure 5E) that classify them as susceptible according to CLSI [25]. They were from T0, T1 or T2 but showed the common characteristic of being of st3 (in one case st5). This contrasted with previous data obtained on French strains (MIC90 > 64 µg/mL for strains collected in 2010–2012 [16]). To further analyze this discrepancy, 33 strains isolated between 2011 and 2018 (mainly st3 and a few st2 as control, see Supplementary Table S1) through the Vigimyc network were selected and their MICs for spectinomycin analyzed (Figure 6). A third of the strains (33.3%) were susceptible to spectinomycin, irrespective of subtype.

No correlation could be found between antimicrobial treatments used in the feedlots (Supplementary Table S1) and either MIC or genetic subtype, whatever the substance used. No selection was observed as a result of the various treatments used in the feedlots.

### 2.4. Antimicrobial Resistance Profile of Pasteurellaceae Is Different from That of M. bovis 

*M. bovis* and Pasteurellaceae were co-isolated in 13 occurrences (five different feedlots, different sampling times) (see Supplementary Table S2 and Figure S1). In these 13 occurrences of co-isolation, the antimicrobial susceptibility profiles of Pasteurellaceae were examined by the disk method. *P. multocida* strains were mainly sensitive to amoxicillin, tulathromycin, tylosin, spectinomycin, florfenicol, enrofloxacin and marbofloxacin but resistant to tetracycline. In one feedlot (CAM) we detected strains resistant to tulathromycin. The sole strain of *M. haemolytica* that was isolated in the study (T1, herd NE) was resistant to amoxicillin, intermediate for enrofloxacin and tulathromycin and susceptible for the other antimicrobials.

In two feedlots (CAS5 and NE), collective antimicrobial treatments could have helped to select resistant strains. In CAS5 where doxycycline was used as metaphylaxis at the BRD peak, isolates were resistant to tetracycline at T1 and T2 but not at T0 (Supplementary Figure S1A,D). In NE, where spectinomycin was used individually, though not for the sampled calves, isolates were resistant to spectinomycin at T1 and T2 but not at T0 (Supplementary Figure S1E).

Pasteurellaceae remained globally largely more susceptible to antimicrobials than *M. bovis*. Although it is hard to conclude on a small number of strains, it seems that treatment influenced Pasteurellaceae antimicrobial susceptibility patterns, as the selection of resistant isolates was observed in two situations.

## 3. Discussion

In the present study, out of 25 monitored feedlots, 21 experienced a BRD episode, of which 18 were positive for *M. bovis*, among other pathogens, at the disease peak (T1). This high prevalence confirms the major contribution of *M. bovis* in BRD in this context of feedlots where animals of several origins are comingled [26]. *M. bovis* was most often associated with other pathogens, such as *P. multocida* and the Coronavirus (Figure 2), also frequently reported in BRD episodes [3,27]. Nevertheless, these prevalence results may be related to our sampling choice, i.e., nasal swabs. The Bovine Coronavirus was indeed shown to be detected in higher proportions in superficial samplings than in the lower respiratory tract samples, such as bronchoalveolar lavages [28]. The overall mycoplasmal load per calf was high at the disease peak, with a mean Ct of 25.7. Nonetheless, in 2/4 feedlots with no BRD episode, we were able to detect *M. bovis*-positive calves at T2, suggesting a potential asymptomatic circulation of the pathogen in the absence of any clinical disease as already suggested [8]. The weak prevalence observed at T0 (2% of positive calves) seems a true picture of the actual circulation of *M. bovis* in dairy herds in France [29], calves reared in feedlots mainly coming from dairy herds.

Four weeks after antimicrobial treatment (T2), when acute clinical signs of BRD were over, 49% of the calves remained rtPCR-positive for *M. bovis* against 51% at T1, indicating failed microbial clearance by treatments. However, the increase in the mean Ct to 30.4 and the low proportion of calves tested positive by culture (19%) suggest that at this stage of clinical recovery, the viable *Mycoplasma* load was significantly reduced. At T2, the proportion of *M. bovis*-positive animals estimated by culture was comparable to that recorded by our epidemiosurveillance network Vigimyc (15%, [18]). This suggests that most often, in day to day diagnosis, mycoplasmas are searched for only after antimicrobial treatment, remaining an etiology explored in case of failure of clinical improvement after chemotherapy. 

We further showed that the overall genetic diversity of strains, assessed by *polC* subtyping [15] and PFGE analysis [30], was unmodified either by the ongoing BRD episode or by the associated antimicrobial treatments. The proportion of the two main *polC* subtypes currently circulating in France was comparable at each sampling time (feedlot entry, BRD peak and 4 weeks after the peak), i.e., 80% st2 and 20% st3. This proportion was also comparable to that of diagnosis strains collected in the framework of the Vigimyc surveillance network [15]. This confirms that our network, in its current operating procedures [18], is able to collect strains representative of those circulating in France and so is a real resource for monitoring genetic diversity and AMR.

PFGE patterns confirmed that st3 strains were more variable than st2 strains [15], but once again, this diversity was not related to any particular evolution of BRD or antimicrobial treatments. Both subtypes could be found circulating in the same herd, although no calf was detected harboring both subtypes at any one time. Considering the marked polymorphisms between st2 and st3, it is unlikely that the switch of subtypes observed on some calves over time (from T1 to T2, st2→st3 or st3→st2) could result from genetic evolution of the strains in such a short interval. The most likely scenario is co-circulation within a lot or a calf of the two subtypes, a possibility not observed here but previously reported and one becoming more prevalent at the time of sampling. Interestingly, a different subtype, never detected before in France, namely st5, was found in the RO feedlot. It had already been described in North America (ST124, in the legacy MLST scheme or ST45 in the revised scheme of Register [31,32]). Further genomic and phylogenetic characterization of this strain is ongoing, especially to establish phylogenetic relationships between the three subtypes.

As expected from studies around the world, our MIC data confirmed the overall multiresistance of *M. bovis* strains. For all the antimicrobials, resistance levels were the same as reported recently elsewhere [12,33,34,35,36,37,38,39,40,41]: *M. bovis* strains were resistant to tetracyclines, macrolides and florfenicol (with a few intermediate strains). However, we were further able to demonstrate that strains were already resistant before any antimicrobial treatment and that their MIC patterns were not changed in the course of the BRD episode and the associated chemotherapy. These results show that resistant clones are not selected during the disease episode but that clones circulating in France are already multiresistant. Fluoroquinolones remain the only antimicrobials with low MICs, which might be due to their restricted use in veterinary medicine owing to their classification as critically important antimicrobials. For this family, although we could fear an MIC increase for st3 due to a greater ability to fix mutations in vitro under subinhibitory concentrations of enrofloxacin [19], we observed the same susceptibility profiles for both subtypes. The few strains showing a slight increase in MIC of enrofloxacin to an intermediate level were st2, most strains of both subtypes being susceptible. The hypothesis of the spread since the year 2000 of a dominant multiresistant clone is thus confirmed [15] and we further rule out the possibility of the co-existence of susceptible clones. 

The situation was different for Pasteurellaceae, for which we were partly able to correlate antimicrobial treatments and change in susceptibility profiles. We managed to gather data on antimicrobial use, although stockbreeders did not always continuously record treatments, which resulted in some incomplete data [40] (Supplementary Table S2). However, potential acquisition of AMR was recorded in two feedlots (CAS5 and NE), where the targeted antimicrobials had been used. This underlines the fact that acquisition of AMR may not have the same dynamics for *M. bovis* and for Pasteurellaceae. For the latter, AMR may arise during the BRD course under the influence of chemotherapy as already demonstrated by the apparition or spreading of new clones [42]. We were able to illustrate this fact only in two herds, because of the difficulty to retrieve Pasteurellaceae from nasal swabs that are often polymicrobial [43].

One unexpected finding in this study was the diversity of susceptibility profiles for spectinomycin, contrasting with previous observations that classified *M. bovis* as 100% resistant to this drug in France [16]. The decrease in spectinomycin MICs in France could signal a reappearance of more susceptible profiles, which are observed elsewhere in the world [33,34]. This mixed situation, with the coexistence of highly and poorly spectinomycin-resistant strains is very similar to what has been described in Hungary [38]. It would be of interest to investigate whether it is associated with a true reversion of antimicrobial resistance genotypes, with notably mutations (and reversions) in the *rrs* genes at position 1192 as previously reported [22,34].

## 4. Materials and Methods

### 4.1. Sampling Campaigns

A total of 537 veal calves were sampled in 25 fattening units located in Western France from November to April 2016–2017 and November to April 2017–2018. The size of each feedlot, ranging from 22 to 519 heads, and the antimicrobial treatments used during the observation periods were recorded (for more details see Supplementary Tables S1 and S2). Figure 1 summarizes the sampling and analysis workflow. In each herd, 10 randomly chosen calves were sampled using double nasal swabs (DNS) when introduced in the feedlot (T0). When a BRD episode occurred in the feedlot and before any collective antimicrobial treatment (T1), DNS were taken from five diseased calves. Four weeks after the end of the collective antimicrobial treatment (T2), the ten calves sampled at T0 were re-sampled, if possible. All DNS (T0, T1, T2) were sent, dry, at 4 °C, within at most two days after sampling, to the Anses laboratory. One swab was used for Pasteurellaceae isolation on Columbia agar plate containing 5% sheep blood (Biomérieux) and then for nucleic acid extraction (see hereafter). Another swab was used for *Mycoplasma* isolation (see below).

### 4.2. Nucleic Acid Extraction from Swabs and rtPCR Amplifications

Nucleic acids were extracted from swabs using the simplified protocol described previously [44]. Briefly, swabs were squeezed and sterilely cut in a tube containing a lysis buffer (TRIS 0.1 M, Tween 20 0.05% and proteinase K 0.24 mg/mL). They were heated for 1 h at 60 °C and then for 15 min at 95 °C to inactivate the proteinase. Real-time PCR (rtPCR) was performed on these bulk extracts using LSI VetMAX Screening pack Ruminant Respiratory Pathogens (ThermoFisher) to detect the various pathogens responsible for BRD (*M. bovis*, *Histophilus somni*, *Pasteurella multocida*, *Mannheimia haemolytica*, Coronavirus, Respiratory Syncytial Virus and Parainfluenza 3) (at T1), or with VetMAX™ *M. bovis* Kit (ThermoFisher) to only assess *M. bovis* presence (at T0 and T2), according to the manufacturer’s recommendations. The Ct cut-off for *M. bovis*-positiveness was set at ≤ 37 according to the recommendation of Wisselink et al. [45].

### 4.3. Isolation and Identification of M. bovis Isolates

Swabs were seeded on plates containing a PPLO agar medium modified as previously described [46], with addition of 0.1% of Tween 80 for specific inhibition of *M. bovirhinis* potential contamination [47]. Plates were incubated for 4 days at 37 °C in 5% CO_2_. A maximum of 10 clones per calf and per sampling point were randomly selected with a wooden toothpick, further cultured in 2 mL PPLO broth and identified using membrane filtration dot-immunobinding tests (MF-Dot) as previously described [48]. As the number of picked clones per calf varied with the quality of the isolation, each calf at one sampling time (T0, T1 or T2) was considered as a single epidemiological unit.

A set of *M. bovis* isolates from a collection kept at Anses, Lyon Laboratory and mostly derived from the French national surveillance network for mycoplasmosis in ruminants (Vigimyc) [18] were included in the study as a “reference” population for both subtyping and antimicrobial resistance.

### 4.4. Strain Subtyping by Sequence Analysis of the Housekeeping Gene polC and Pulse Field Gel Electrophoresis (PFGE)

Genomic DNA was extracted from 200 µL of each clone culture using QIAamp^®^ DNA Minikit (Qiagen), and the *M. bovis* clones were all subtyped using *polC* sequence analysis as previously described [15] (Figure 1).

One isolate yielded a new *polC* subtype and was further analyzed to determine its subtype according to the MLST scheme of Register et al. ([24]; https://pulmlst.org/bovis/), by a whole genome resequencing approach. Briefly, a DNA sample was sequenced using an Illumina MiSeq technology generating 2 × 300-bp pair-end reads (MiSeq 600 cycles V3 kit, Biofidal, Vaux-en-Velin, France). A total of 1,443,759 reads were generated for each R*, resulting in an average coverage of 860 X. Trimmed reads (using Trimmomatic-0.36) were aligned to two reference genomes, namely PG45 (refseq NC_014760.1) and JF4278 (a corrected version provided by Bern University). 

After quality control of the alignments, the variants were identified and annotated using GATK4 v4.0.10.0 (https://software.broadinstitute.org/gatk). The filtered output vcf files were then used to retrieve the different Register loci sequences [24].

A sub-panel of clones was selected (one st2 per calf at each sampling time and per lot and all st3, Figure 1 and Supplementary Table S1) to be further subtyped by Pulse-Field Gel Electrophoresis (PFGE) with the *MluI* enzyme as previously described [30]. Briefly, mycoplasma cells from overnight cultures were embedded in low melt agarose plugs and lysed by proteinase K before DNA overnight restriction using endonucleases. The macrorestriction fragments were separated by electrophoresis on a CHEF-DR III system (Bio-Rad) in 1% agarose gel, in TBE 0.5% at 14 °C, for 24h, with an included angle of 120°. Images were analyzed with the software Bionumerics GelCompar II v6.6 (Applied Maths NV, Sint-Martens-Latem, Belgium). The similarity analysis was carried out using the Dice coefficient (position tolerance 1.5%) and a dendrogram was constructed using the UPGMA method.

### 4.5. Antimicrobial Susceptibility Testing of M. bovis and Pasteurellaceae

The susceptibility of the selected *M. bovis* clones was tested using Minimum inhibitory concentration (MICs) assays as previously described [17] for the five antimicrobial classes mostly used to treat BRD in the field and known to be potentially active against *Mycoplasma* spp: quinolones (enrofloxacin), tetracyclines (oxytetracycline), phenicols (florfenicol), aminoglycosides (spectinomycin) and macrolides (tilmicosin). Briefly, clones were plated on PPLO agar plates containing twofold increasing antimicrobial concentrations, either as a full range of antimicrobial dilutions or only for a few concentrations corresponding to the CLSI clinical breakpoints for Pasteurellaceae, a family known to colonize the same body niche [25]. At least two experiments were conducted, and the modes of the different results were retained as the final MIC values. For some strains, the different experiments did not allow us to conclude on a single MIC value: an MIC interval was defined (Supplementary Table S2) but was not represented in Figure 5.

Antibiograms for Pasteurellaceae were outsourced at the Laboratoire Vétérinaire Départemental du Rhône. For each sample positive for both *M. bovis* and Pasteurellaceae, a mix of Pasteurellaceae-like colonies with similar phenotype was collected from the Columbia agar plate, identified with an API 20E gallery and tested for resistance with the standardized diffusion method in agar (norm NF U47-107) for the same five antimicrobial classes tested for *M. bovis* (depending on the available disks): quinolones (enrofloxacin and marbofloxacin), tetracyclines (tetracycline), phenicols (florfenicol), aminoglycosides (spectinomycin) and macrolides (tylosin and tulathromycin). The β-lactam amoxicillin known to be active against Pasteurellaceae was also tested. Zone diameters were interpreted according to the CLSI standards [25].

## 5. Conclusions

This study demonstrates that *M. bovis* is an important player in feedlot BRD. Its prevalence is weak at entry but rapidly increases to reach a peak at the disease onset. It can circulate in the absence of clinical episodes and remain present even after antimicrobial treatments, which can result in clinical recovery without mycoplasmal clearance. The disease course and the associated chemotherapy did not affect the genetic diversity or AMR patterns of strains circulating in a lot. The strains observed in this longitudinal study reflected the general population circulating in France, with one major clone multiresistant to the main antimicrobials used in BRD, also retrieved by our surveillance network.

## Figures and Tables

**Table 1:** Number of double nasal swabs (DNS), calves harboring *M. bovis* (assessed with culture and real-time Polymerase Chain Reaction (rtPCR)) and clones isolated at each sampling time.

	T0	T1	T2	Total
DNS	271	115	151	537
Calves with *M. bovis* rtPCR	6 (2%)	59 (51%)	39/79 * (49%)	104
Calves with *M. bovis* culture	5 (2%)	60 (52%)	28 (19%)	93
Isolated clones	38	251	125	414

T0, feedlot entry; T1, BRD onset; T2, 4 weeks after antimicrobial treatment. * Not all T2 samples were tested by rtPCR (see Supplementary Table S1 for details).

## Supplementary Materials

The following are available online at https://www.mdpi.com/2076-0817/9/7/593/s1, Figure S1: Comparative evolution of zone diameters for the Pasteurellaceae strains before (T1) and 4 weeks after antimicrobial treatment (T2). Table S1: Characteristics of the feedlots. Table S2: Characteristics of the clones studied.
